# Myxopapillary ependymoma with interval postoperative CSF seeding: A report of an unusual case

**DOI:** 10.1016/j.radcr.2021.09.022

**Published:** 2021-10-10

**Authors:** Ali M Abdu, Sultan A Alshoabi, Abdulbaset M Alshoaibi, Abdullgabbar M Hamid, Miral D Jhaveri

**Affiliations:** a Radiology Unit, King Saud University Medical City, Riyadh, Kingdom of Saudi Arabia; b College of Applied Medical Sciences, Taibah University, Almadinah, Kingdom of Saudi Arabia; c Radiology Department, Prince Mohammed Bin Abdulaziz Hospital, Riyadh, Kingdom of Saudi Arabia; d Radiology Department, Rush University Medical Center, Chicago, Illinois, USA

**Keywords:** Myxopapillary ependymoma. Interval CSF seeding, MPE, myxopapillary ependymoma, WHO, world Health Organization, MRI, magnetic resonance imaging, SI, signal intensity, WIs, weighted images, L2, second lumbar vertebra, GTR, gross total resection, T3, third thoracic vertebra, T4, fourth thoracic vertebra, CSF, cerebrospinal fluid°C degree centigrade, kg, kilogram, CNS, central nervous system

## Abstract

Myxopapillary ependymoma (MPE) is a unique slow-growing benign (WHO grade 1) subtype of spinal cord ependymoma arising predominantly in the filum terminale. Despite its benign nature, it occasionally disseminates through the cerebrospinal fluid and metastasizes to distant sites. Here, we report an extremely rare case of MPE with interval CSF seeding and metachronous metastasis in a 47 –year-old female presented as a gradually increasing low back pain for three months with bilateral radiculopathy down to the knees. Magnetic resonance imaging (MRI) showed an intradural extramedullary spinal mass of iso-intense signal to the cord on T1 weighted-images (WIs), heterogeneous, predominantly hyperintense signal on T2WIs with homogenous enhancement after contrast administration. L2 laminectomy with gross total resection (GTR) was performed, and histopathological results confirmed the diagnosis of MPE. Adjuvant radiotherapy was administered, followed by series of MRI scans. 28 months after GTR, Lumbar MRI showed multiple tiny enhancing nodules in the cauda equina. 44 months follow-up whole spine MRI revealed multiple intradural extramedullary nodules throughout the entire spine. The largest one measures about 1.5cm opposite to T3 –T4 intervertebral disc space. The patient underwent T3 and T4 laminectomy and GTR under general anesthesia using microsurgical techniques, and the histopathological result came with the diagnosis of MPE.

## Introduction

Myxopapillary ependymoma (MPE) is a distinct slow-growing benign (WHO grade 1) subtype of spinal cord ependymoma that likely arises from the ependymal cells in the filum terminale. It is the most common primary neoplasm of the conus medullaris and cauda equina [1, 2]. MPE represents approximately 13% of spinal ependymomas and 90% of the conus medullaris tumors [3]. It is usually an intradural extramedullary tumor and almost exclusively presents near the conus medullaris and filum terminale. Despite the benign nature of the tumor, it can exhibit local recurrence or bleeding into the cerebrospinal fluid (CSF), leading to CSF seeding and spread into other regions of the central nervous system (CNS) [2, 4]. Here, we report a case of MPE presented with CSF seeding after several months of total surgical excision. The magnetic resonance imaging (MRI) and histopathologic features of MPE are described, along with a discussion of CSF seeding of the tumor.

## Case report

**Fig. 1:**
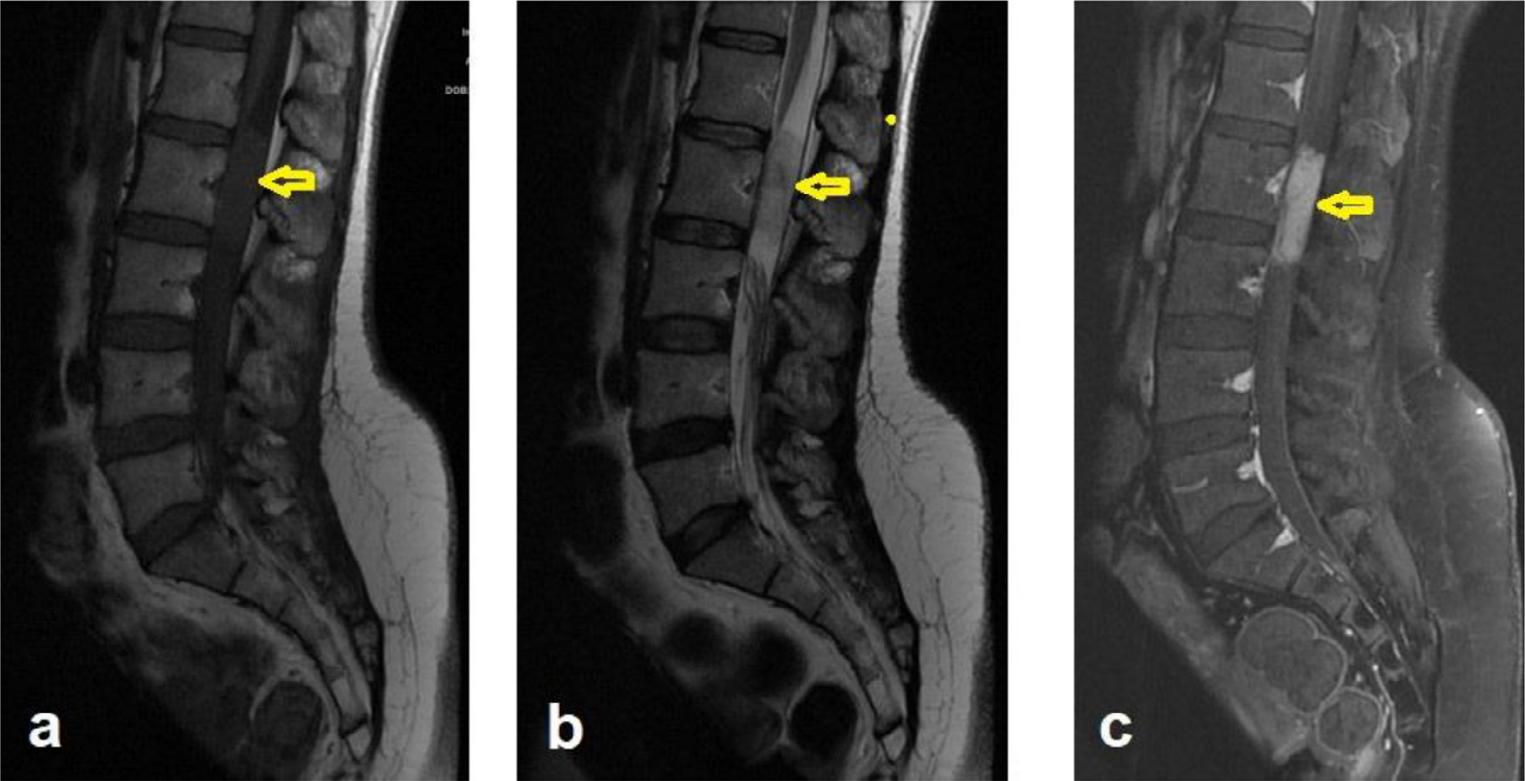
Sagittal MRI of the lumbar spine demonstrates an extramedullary, intradural spinal mass centered at L2 level. The lesion is low/isointense on T1 (A), heterogenous signal on T2 (B), and homogenously enhancing after contrast administration (C)

A 47 –year-old female presented with gradually increasing low back pain for three months with bilateral radiculopathy down to the level of the knees more on the left side. No sphincteric problems, fever, or other complaints. On examination: the patient was conscious, oriented with normal upper and lower limb tone, sensation, and reflexes. Lumbosacral MRI showed an extramedullary, intradural spinal mass at the L2 level that demonstrated an iso-intense signal to the cord on T1 weighted-images (T1WIs), heterogeneous, predominantly high signal on T2-weighted images (T2WIs), and homogenous enhancement after contrast administration. The patient underwent surgical resection with an L2 laminectomy and gross total resection (GTR). Histopathological results were consistent with MPE (WHO grade 1) (Fig. 1). Adjuvant radiotherapy was administered.

**Postoperative course:** The patient was doing well without any neurologic deficits. Postoperative follow-up MRI confirmed GTR of the neoplasm with no residual tumor.

**Fig. 2:**
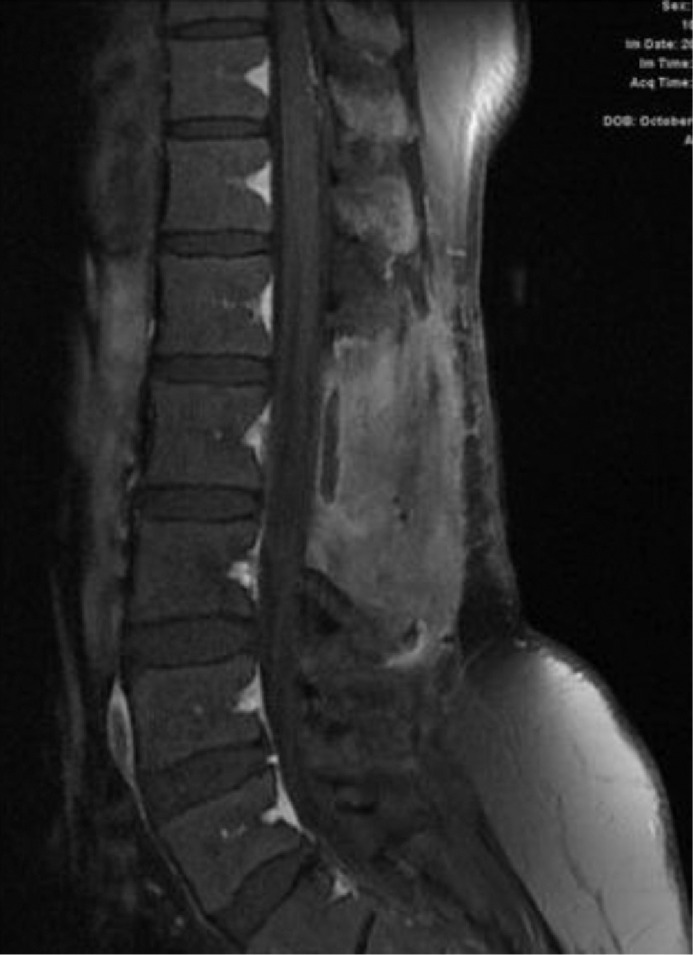
T1 post-contrast image eight months following surgery show post-surgical changes with no residual or recurrence tissue of the tumor

**8 months after GTR,** follow-up lumbosacral MRI showed no detectable tumor recurrence (Fig. 2).

**Fig. 3:**
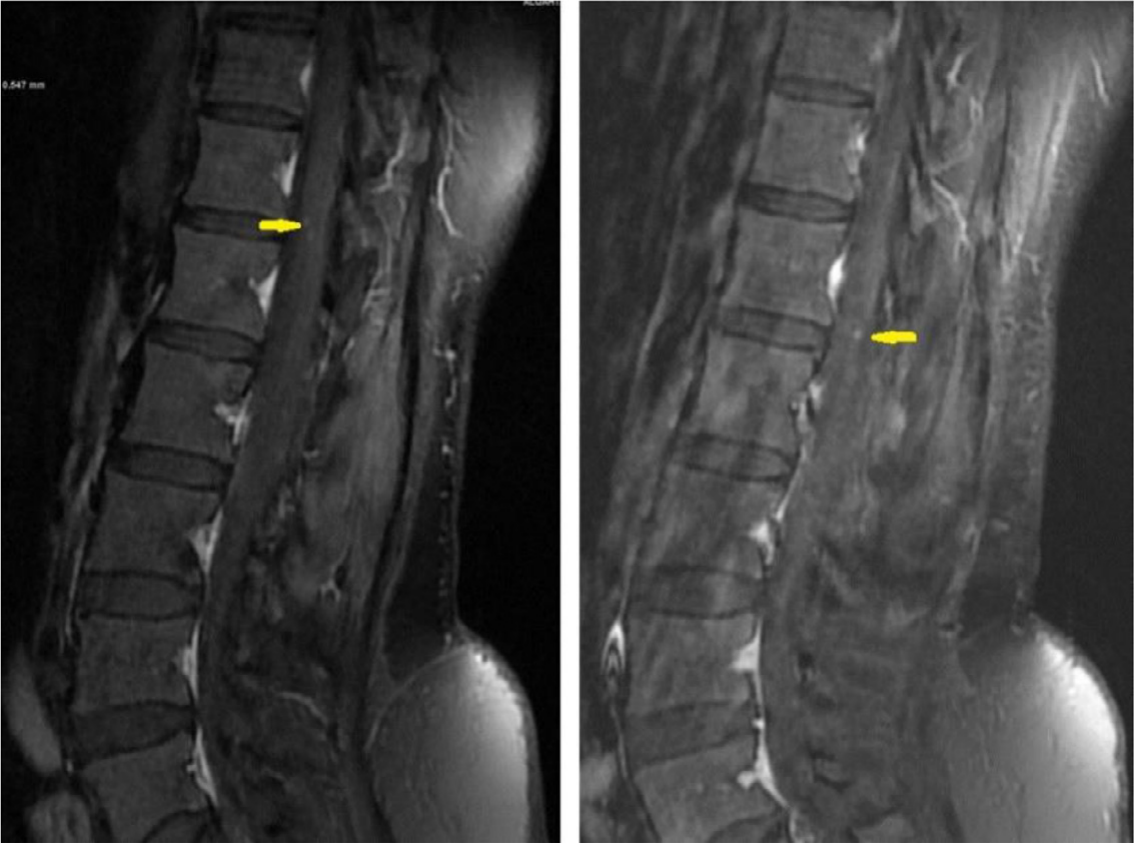
MRI lumbar spine 28 months after surgery show tiny enhancing nodules in the cauda equina consistent with CSF seeding (arrows).

**28 months after GTR,** follow-up lumbosacral MRI showed tiny enhancing nodules in the cauda equina, but the patient was free of symptoms (Fig. 3).

**Fig. 4:**
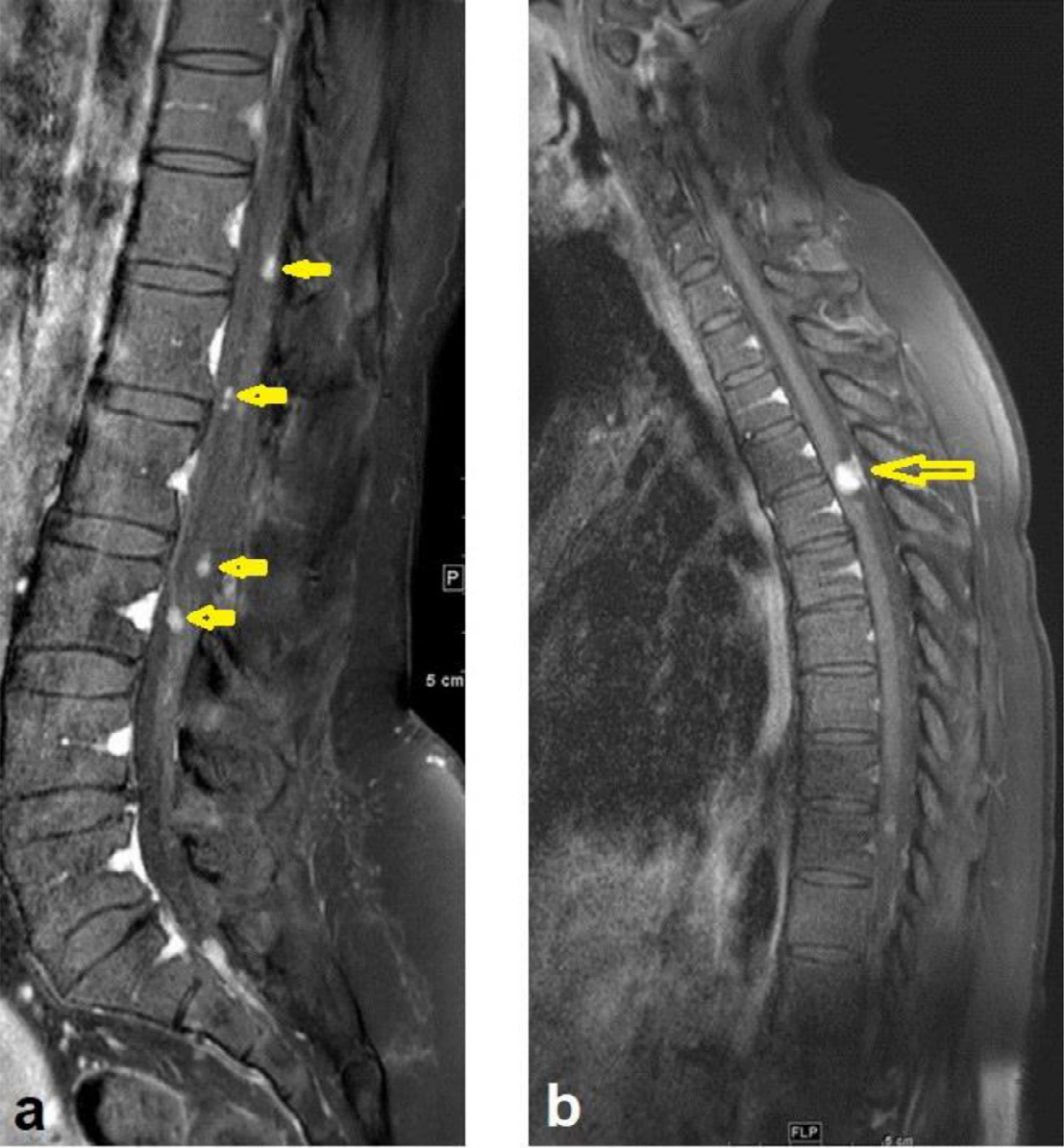
T1 postcontrast sagittal images 44-months post-surgery: (A) Lumbar spine with multiple new enhancing nodules along the cauda equina (arrows) and (B) Thoracic spine shows a well-defined intradural extramedullary lesion at the level of T3/T4 with cord compression (arrow)

**44 months after GTR,** the patient presented with 2 months of back pain, progressive right lower limb numbness, and urine incontinence. No fever, lower limb weakness, or other symptoms. On examination, the patient was conscious and oriented but with impaired tandem gait. The lower limb power was 5/5 in all joints except the right hip joint, which was 4/5. Whole spine MRI revealed multiple intradural extramedullary nodules throughout the spine. The largest one measures about 1.5cm at T3 –T4 level (Fig. 4B). The patient planned for surgical resection. GTR was performed using microsurgical techniques. Samples were sent to histopathology for examination, and the results confirmed the diagnosis of MPE (WHO grade 1).

**Fig. 5:**
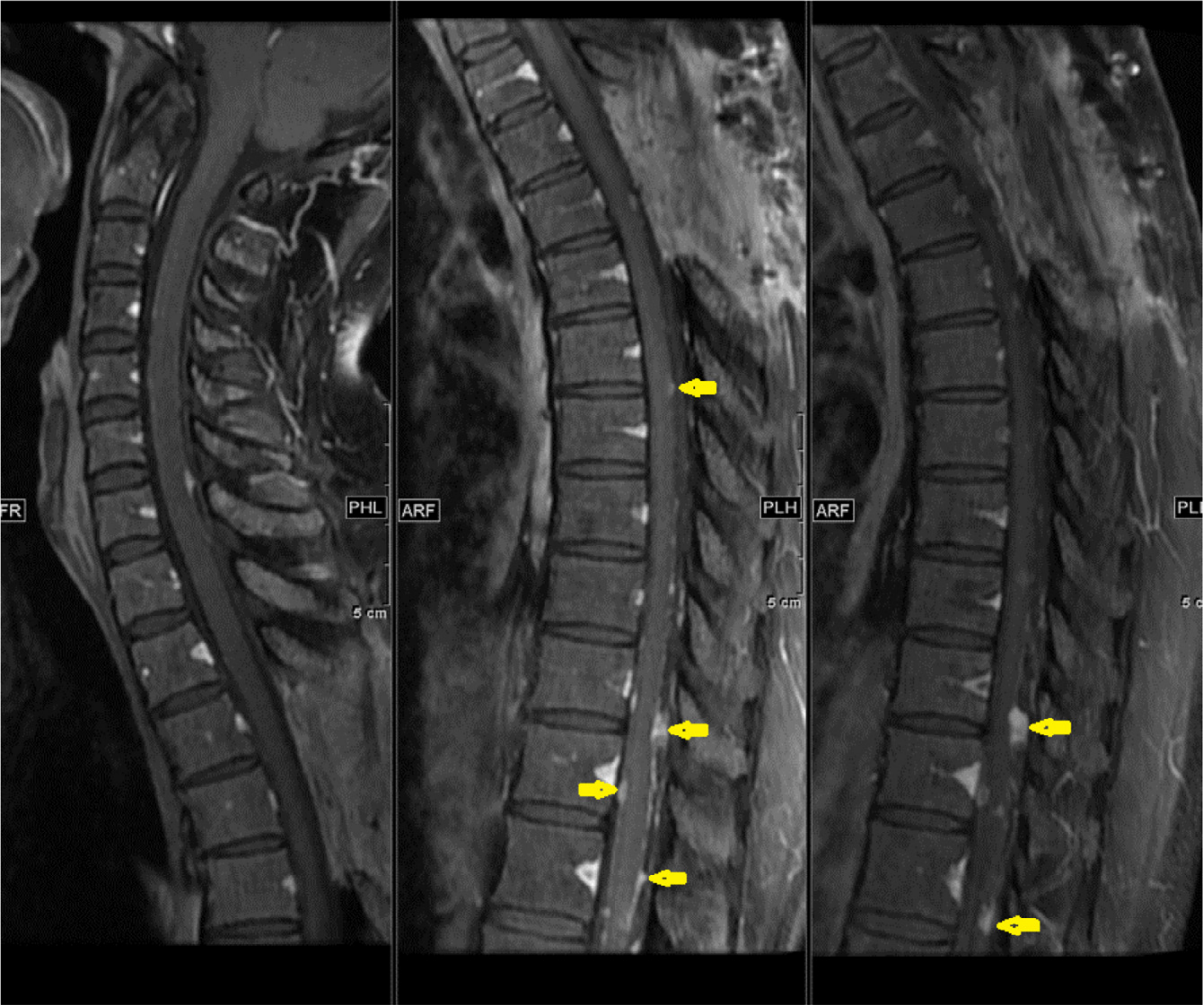
Sagittal MRI images of cervical and thoracic spine 48-months post-surgery show post-surgical changes in the upper thoracic spine following resection. Enlarging and new enhancing nodules consistent with CSF seeding (arrows)

**Fig. 6:**
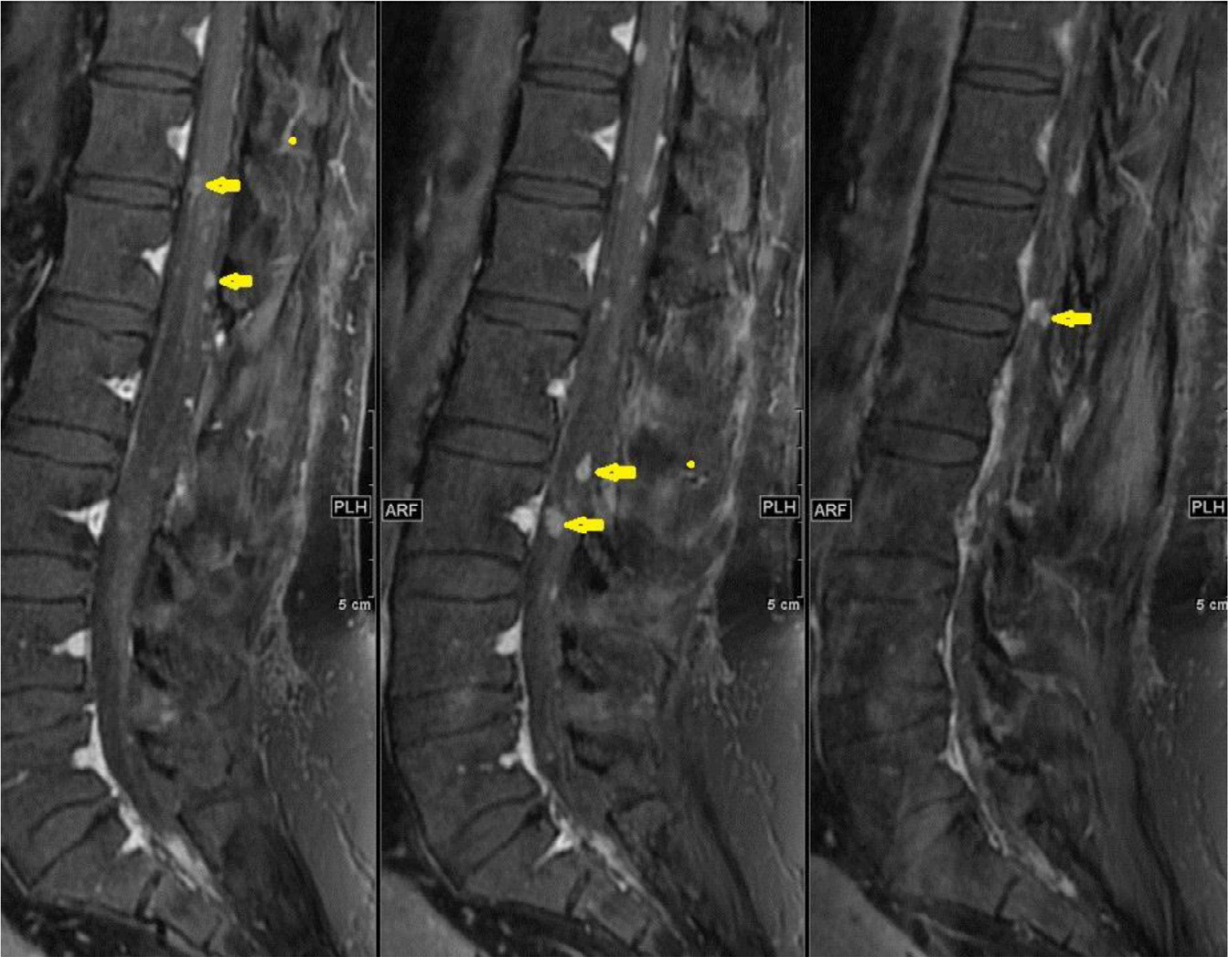
Sagittal MR images 48-months post-surgery of lumbar spine show increased number and size of the multiple enhancing intradural extramedullary nodules (arrows)

**Fig. 7:**
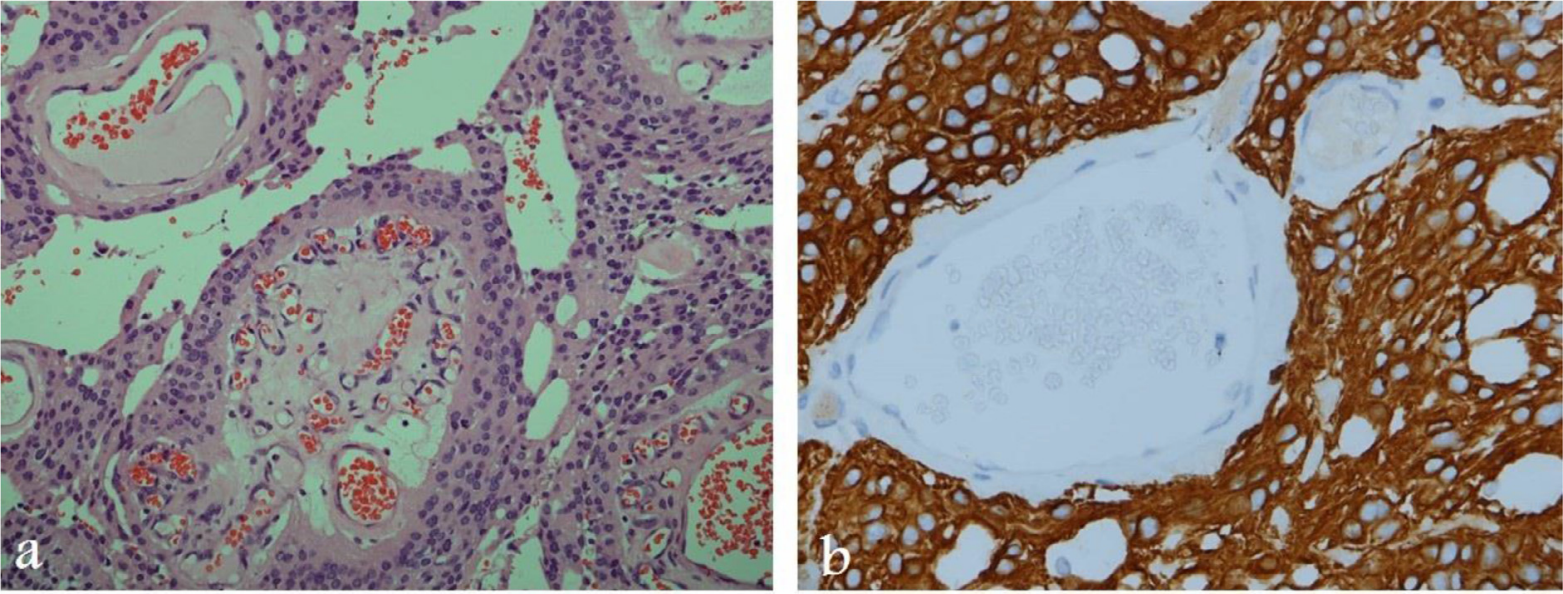
Selected images of histopathology examination:( A) Hematoxylin and eosin stain (H&E stain x 200) of lower power microscopic view shows hyalinised vascular papillary cores with surrounding ependymal cells, (B) Immunohistochemically stain for glial fibrillary acidic protein (GFAP stain x 400) shows strong positive staining and confirming the diagnosis of ependymoma

**At subsequent follow-up four months after the second surgery,** the patient's numbness and urinary symptoms improved. A follow-up MRI showed surgical wound healing (Fig. 5) and multiple enhancing CSF seedings (Fig. 5 and 6). A biopsy was taken, and histopathology examination confirmed the diagnosis of MPE (Fig. 7).

## Discussion

MPE is a distinct subtype of spinal cord ependymoma that usually presents as an intradural tumor almost exclusively near the conus medullaris and filum terminale. Although a benign tumor, it can exhibit local recurrence or CSF spread into other regions of the CNS.

Cachia et al. [5] reported that MPE can be large enough to fill the spinal canal and may present lower back pain and symptoms of nerve root compression, as seen in our case. MPE often appears as a well-defined ovoid or sausage-shaped mass isointense to the spinal cord on T1WIs with avid enhancement after contrast administration and high SI on T2WIs with or without hypointense margins of hemosiderin deposition.

Despite the WHO grade 1 classification of MPE, there are few reports of primary CSF dissemination. Khan et al. reported a case of MPE with primary CSF seeding [6]. CSF seeding is rarely seen in adults with MPE, which could be a sign of aggressive behavior. The current case showed CSF seeding scattered in the spine with subsequent second surgical intervention in the thoracic spine for a compressing lesion presenting with lower limb weakness and urinary incontinence diagnosed 44-months after the first surgery.

Similar cases of MPE presenting with cauda equina symptoms secondary to CSF seeding presenting several months after primary surgery and adjuvant radiotherapy were reported by Rege et al. [7] and Bates et al. [8]. the latter case was subjected to second surgery for metastasis resection.

Regarding the treatment of MPE, a literature review showed that GTR of the neoplasm is the optimal primary choice [7], [8], [9]. However, GTR was not effective in our case, and CSF seeding was reported 28 months after the primary surgery with continual progression. Several studies suggested adjuvant radiotherapy to accentuate MPE local control and CSF seeding free survival [8.9]. But again, this was not effective in our case.

An interesting case with striking similarities with ours was reported by Huynh et al. [10] in a 24-year-old female.

## Conclusion

MPE is a benign type of ependymoma that has a potential for local recurrence and CSF seeding even after gross total resection and adjuvant radiotherapy. This behavior is extremely rare but base-line whole spine MRI to detect synchronous CSF seedings appears reasonable, likewise, follow-up imaging, mainly in case of recurrent symptoms.

## Patient consent

Informed consent was obtained from the patient to publish this case report.
